# Oxidative-stress induced increase in circulating fatty acids does not contribute to phospholipase A_2_-dependent appetitive long-term memory failure in the pond snail *Lymnaeastagnalis*

**DOI:** 10.1186/1471-2202-15-56

**Published:** 2014-05-01

**Authors:** Emily Beaulieu, Julie Ioffe, Shawn N Watson, Petra M Hermann, Willem C Wildering

**Affiliations:** 1 Department of Biological Sciences, Faculty of Science, University of Calgary, Calgary, Alberta T2N 1N4, Canada; 2 Department of Physiology and Pharmacology, Faculty of Medicine, Hotchkiss Brain Institute, University of Calgary, Calgary, Alberta T2N 4N1, Canada

**Keywords:** Cognitive impairment, Lipid peroxidation, Classical conditioning, Invertebrate, Phospholipase A2, Arachidonic acid, Mollusc, Oxidative stress, Free fatty acid

## Abstract

**Background:**

Reactive oxygen species (ROS) are essential for normal physiological functioning of the brain. However, uncompensated increase in ROS levels may results in oxidative stress. Phospholipase A_2_ (PLA_2_) is one of the key players activated by elevated ROS levels resulting in the hydrolysis of various products from the plasmamembrane such as peroxidized fatty acids. Free fatty acids (FFAs) and fatty acid metabolites are often implicated to the genesis of cognitive impairment. Previously we have shown that age-, and experimentally induced oxidative stress causes PLA_2_-dependent long-term memory (LTM) failure in an aversive operant conditioning model in *Lymnaea stagnalis*. In the present study, we investigate the effects of experimentally induced oxidative stress and the role of elevated levels of circulating FFAs on LTM function using a non-aversive appetitive classical conditioning paradigm.

**Results:**

We show that intracoelomic injection of exogenous PLA_2_ or pro-oxidant induced PLA_2_ activation negatively affects LTM performance in our learning paradigm. In addition, we show that experimental induction of oxidative stress causes significant temporal changes in circulating FFA levels. Importantly, the time of training coincides with the peak of this change in lipid metabolism. However, intracoelomic injection with exogenous arachidonic acid, one of the main FFAs released by PLA_2_, does not affect LTM function. Moreover, sequestrating circulating FFAs with the aid of bovine serum albumin does not rescue pro-oxidant induced appetitive LTM failure.

**Conclusions:**

Our data substantiates previous evidence linking lipid peroxidation and PLA_2_ activation to age- and oxidative stress-related cognitive impairment, neuronal dysfunction and disease. In addition however, our data indicate that lipid peroxidation induced increased levels of circulating (per)oxidized FFAs are not a factor in oxidative stress induced LTM impairment.

## Background

Reactive oxygen species (ROS) play an important and critical role in synaptic plasticity and memory formation [1,2]. However, high levels of ROS are also shown to cause detrimental effects in the brain that negatively affect neuronal plasticity and memory function [3,4]. For instance, excessive (experimentally) increased levels of ROS are associated with decreased cognitive performance in both vertebrate and invertebrate species [1,2,4-7]. Under normal physiological conditions, the potential damaging effects of ROS is controlled by robust antioxidant defense systems. However, excessive formation of free radicals due to for instance an inflammatory reaction or failing antioxidant capacity as observed in aging, might overwhelm the antioxidant defense capacity of an organism resulting in oxidative stress. Oxidative stress may cause structural damage to nucleotides, lipids and proteins. The neuronal plasmamembrane is particularly prone to oxidative insult due to its high poly-unsaturated fatty acid (PUFA) content [8-11]. Many processes participate in the repair of oxidative damage to cellular components. In the case of membrane phospholipids, members of the Phospholipase A_2_ (PLA_2_) family of fatty acid acylases that catalyze hydrolysis of *sn-2* lipid bonds in glycerophospholipids are key players [12,13]. PLA_2_’s promote the formation of lysophopholipids and free fatty acids (FFAs) such as arachidonic acid (AA) and docosahexaenoic acid (DHA) [14-16]. Under normal physiological conditions PLA_2_, its products and their metabolites play essential roles in regulating signal transduction, various neuronal signaling pathways, ion channel functioning and gene transcription processes [15,17-21]. Under conditions of oxidative stress PLA_2_ is one of the mediators through which peroxidized fatty acids are excised from the lipid bilayer matrix [12,15]. Recent evidence increasingly associates deregulation of lipid metabolism due to PLA_2_ (over) activation as a cause of nervous system dysfunction and cognitive impairment [7,12,22,23]. Yet, the physiological mechanisms underlying these phenomena remain incompletely resolved.

Our current research on the foundations of learning and memory impairment in the pond snail *Lymnaea stagnalis* (*L. stagnalis; L.*) focuses on the question of lipid-peroxidation dependent facets of neuronal dysfunction [6,7,22]. The present study follows from our recent observations implicating age-, inflammation- and experimental oxidative stress-induced PLA_2_ activity in long-term memory (LTM) failure [7,22]. Considering the pivotal role of PLA_2_ activation in behavioural plasticity, we investigated the question whether it is the fatty acids released upon PLA_2_ activation that causes cognitive impairment in our model system. Here we utilized an established and widely studied classical appetitive reward-conditioning paradigm involving chemosensory conditioning of the animals’ feeding behaviour i.e., “rasping” [22,24-27]. Using this model we investigated the effects of experimentally pro-oxidant induced PLA_2_ activation on associative appetitive LTM impairment and the role of circulating FFAs therein. We will provide evidence that increased levels of circulating (per)oxidized fatty acids are not a factor in oxidative stress induced LTM impairment.

## Results

### Enhanced levels of extracellular PLA_2_ inhibit LTM formation

**Figure 1:**
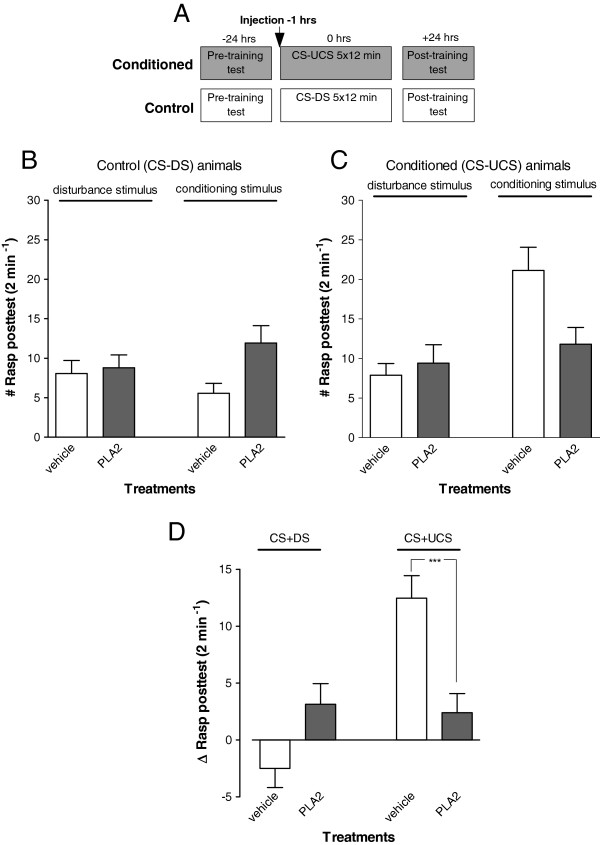
**Long-term memory assessments after bee venom PLA**_**2 **_**injection. A**. Protocol indicating timing of injection and start of pre- and post-training tests with respect to the start of the first training session for both the conditioned (CS-UCS) and control (CS-DS) animals. **B-D** LTM assessment in animals injected 1 hr before training. In all control animals, rasping movements were similar upon application of the disturbance stimulus or conditioning stimulus **(B)**. In contrast, in conditioned vehicle injected animals the number of rasping movements increased after application of the conditioning stimulus **(C)**. The difference between the number of rasps induced by the conditioning stimulus minus the disturbance stimulus shows that there was a robust response to conditioning but only in the vehicle injected animals. None of the unconditioned animals or the PLA_2_ injected conditioned animals responded with significant feeding movements in the post-training test **(D)**. These results indicate LTM impairment in snails injected with exogenous PLA_2_ 1 hr before training. ***p < 0.001.

To assess the impact of increased levels of extracellular PLA_2_ on the formation of appetitive LTM, animals randomly assigned to two test groups were injected with PLA_2_ from bee venom or vehicle-only 1 hr before their first training session. Animals were subsequently tested for the presence of conditioned feeding responses 22–24 hrs after their last training session (Figure 1A). These experiments revealed a prominent suppression of conditioned feeding responses in animals that received treatment with PLA_2_ (Figure 1B; ANOVA interaction time x treatment; F_1,57_ = 19.315, p < 0.0001). That is, vehicle-injected conditioned (CS-UCS) animals displayed robust conditioned feeding responses compared to their non-conditioned (CS-DS) peers (F_1,57_ = 35.672 p < 0.0001). In contrast, PLA_2_-injected animals displayed a conditioned feeding response that was significantly reduced compared to the vehicle conditioned animals (F_1,57_ = 15.634 p = 0.0002) and was statistically indistinguishable from their non-conditioned partners (F_1,57_ = 0.083, p = 0.77).

### AAPH injection elevates circulating FFA levels and causes PLA_2_-dependent LTM failure

**Figure 2:**
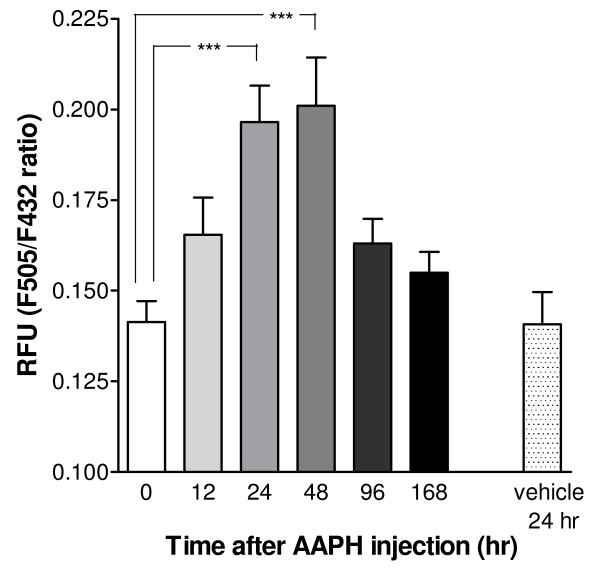
**Temporal characteristics of AAPH induced haemolymph free fatty acid levels.** Average ADIFAB F_505_/F_432_ relative fluorescence intensity ratios (RFU) measured in haemolymph samples collected 24 hrs after vehicle injection and immediately or 12, 24, 48, 96, and 168 hrs after intracoelomic injection with AAPH. There is a substantial and significant increase in F_505_/F_432_ ratio when measured 24 hrs and 48 hrs after AAPH injection but not after vehicle injection. *** p < 0.001.

Prior studies in *L. stagnalis* implicate natural and experimentally (AAPH) induced non-enzymatic lipid peroxidation to memory deficiencies in an aversive operant conditioning of aerial respiratory behaviour [7]. We also demonstrated this phenomenon can be fully reversed through treatment with the general PLA_2_ inhibitor aristolochic acid [7]. Therefore, we now proceed with testing whether AAPH treatment also affect non-aversive appetitive classical conditioning. To this end, we first evaluated the temporal characteristics of AAPH induced free fatty acid (FFA) release into the circulatory system. FFA content of haemolymph extracted immediately or 12, 24, 48, 96 and 168 hours after intracoelomic injection of AAPH were quantified with ADIFAB-FFA assay. A significant increase in haemolymph FFA levels developed over the first 24–48 hours after AAPH injection (Figure 2; One-way ANOVA F_6,44_ = 7.798, p < 0.0001; Planned comparison 0 hr vs 24 hrs F_1,44_ = 21.538, p < 0.0001 and 0 hr vs 48 hrs F_1,44_ = 23.536, p < 0.0001). No such increase was observed when haemolymph was collected 24 hrs after vehicle injection (Planned comparison 0 hr vs vehicle 24 hrs F_1,44_ = 0.0025, p = 0.96). FFA levels returned to control levels 2–4 days after AAPH injection. These data support the notion that AAPH injection can cause significant temporal changes in lipid metabolism within the whole animal.

**Figure 3:**
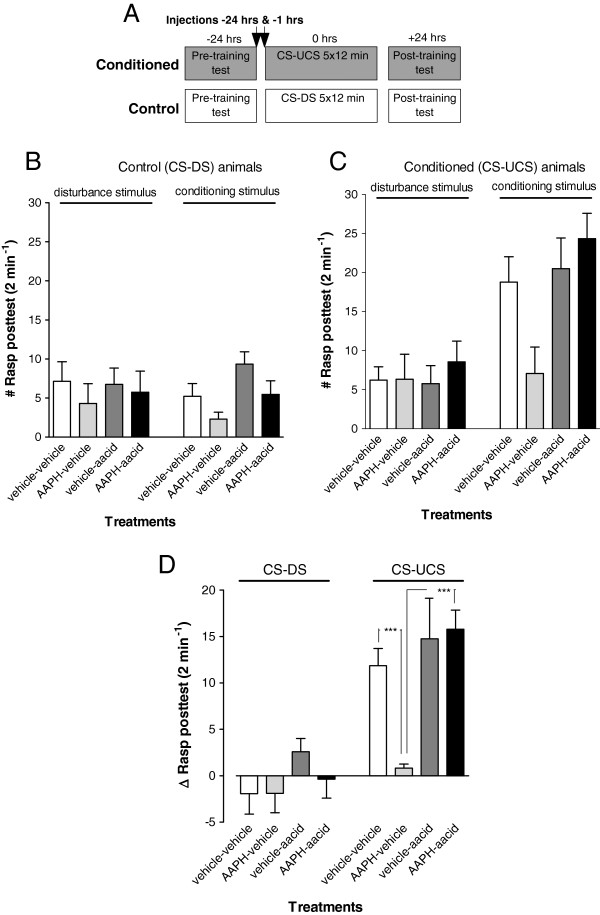
**PLA**_**2 **_**inhibition restores LTM deficiency in AAPH injected animals. A**. Protocol indicating timing of injection and start of pre- and post-training tests with respect to the start of the first training session for both the conditioned (CS-UCS) and control (CS-DS) animals. **B-D**. LTM assessment in animals injected with either vehicle only (vehicle-vehicle), AAPH only (AAPH-vehicle), aristolochic acid only (vehicle-aacid) or injected with both AAPH and aristolochic acid (AAPH-aacid). In all control animals, rasping movements were similar upon application of the disturbance stimulus or conditioning stimulus **(B)**. In contrast, conditioned animals, either vehicle injected, aristolochic acid only or AAPH + aristolochic acid injected, showed an increase in the number of rasping movements after application of the conditioning stimulus **(C)**. Thus, the AAPH injected conditioned group, showed a significant reduction in their Δrasp values in the post-training test compared to all other conditioned groups. Animals injected with aristolochic acid only or AAPH + aristolochic acid were not different in their conditioned response than the vehicle injected animals **(D)**. This suggests that co-treatment of AAPH with a general PLA_2_ inhibitor reverses the AAPH induced adverse effect on appetitive LTM performance. ***p < 0.001.

Next we tested the effect of AAPH induced oxidative stress on non-aversive appetitive classical conditioning and the involvement of PLA_2_ in this process. To this end animals were injected 24 hrs before the first training trial with either vehicle or AAPH. Subsequently, animals were injected 1 hr before the start of the first training session with either aristolochic acid or vehicle only (Figure 3A). We found that treatment with AAPH caused a significant reduction in the conditioned feeding response and that treatment with the PLA_2_ inhibitor aristolochic acid counteracted AAPH’s negative effect on LTM formation (Figure 3B; ANOVA interaction training x treatment, F_3,85_ = 2.847, p < 0.05). That is, AAPH treated animals had a lowered feeding response compared to vehicle treated conditioned animals (F_1,85_ = 11.668, p < 0.001) and their response was statistically indistinguishable from the non-conditioned peers (Figure 3B; F_1,85_ = 0.6016, p = 0.44). In contrast animals treated with aristolochic acid only learned well (F_1,85_ = 13.805, p = 0.0003) in a manner that was statistically indistinguishable from that of vehicle treated animals (Figure 3B; F_1,85_ = 0.841, p = 0.36). Moreover, animals treated with the combination of AAPH and aristolochic acid did display a significant conditioned feeding response (Figure 3B black bars; F_1,85_ = 20.047, p < 0.0001) that was significantly different from the AAPH injected animals (Figure 3B; F_1,85_ = 17.219, p < 0.0001). Note that training and testing trials were performed when AAPH-induced haemolymph FFA levels were at or close to their peak.

### Arachidonic acid does not affect LTM performance

**Figure 4:**
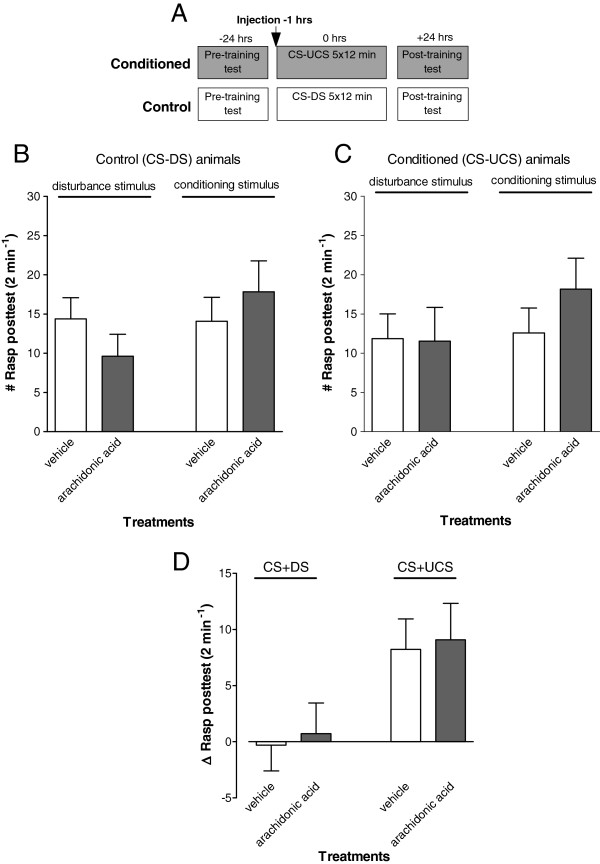
**Long-term memory assessment after injection with arachidonic acid. A**. Protocol indicating timing of injection and start of pre- and post-training tests with respect to the start of the first training session for both the conditioned (CS-UCS) and control (CS-DS) animals. **B-D**. LTM assessment in animals injected 1 hr before training. There was a robust response to conditioning in both vehicle and arachidonic acid injected animals. Thus all conditioned animals responded with significant feeding movements in the post-training test upon application of the conditioning stimulus. These results indicate that injection with exogenous arachidonic acid does not induce LTM impairment in snails.

The above results clearly implicate PLA_2_ as a critical factor in oxidative stress induced appetitive LTM deficiency. PLA_2_ is responsible for the hydrolysis of membrane phospholipids resulting in the release of FFAs including arachidonic acid as one of its most prominent products. Arachidonic acid is the primary substrate for the cyclooxygenase (COX) branch of the eicosanoid pathway. We previously showed that systemic immune challenges cause oxidative stress and induces a PLA_2_- and COX-dependent appetitive LTM failure in *L. stagnalis*[22]. Thus, to test the hypothesis that elevated haemolymph arachidonic acid levels underlie LTM impairment, animals were injected with exogenous arachidonic acid or vehicle-only before their first training session and tested for the presence of conditioned feeding responses 22–24 hrs after their last training session (Figure 4A). These experiments revealed no significant negative effect of arachidonic acid injection on appetitive LTM (ANOVA interaction time x treatment; F_1,48_ = 0.001, p = 0.99; planned comparison treatment vs training F_1,48_ = 9.4584, p = 0.003).

### Binding of free fatty acids to BSA does not rescue LTM failure

**Figure 5:**
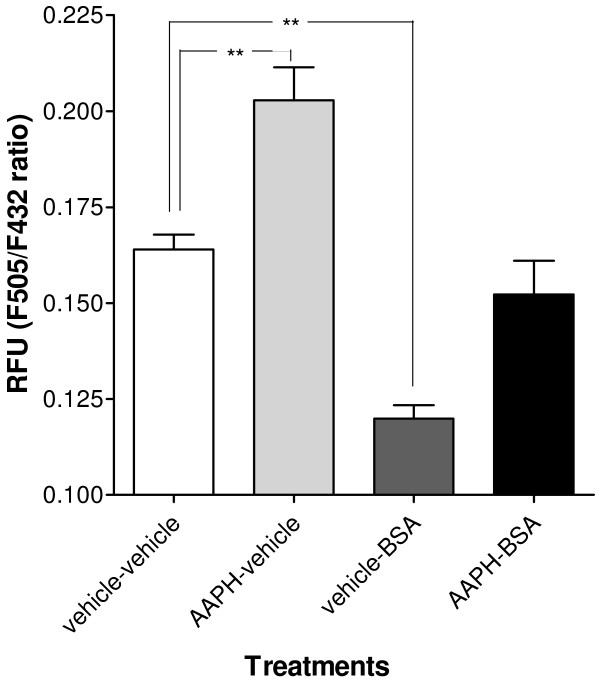
**BSA binds circulatory free fatty acids.** Average ADIFAB F_505_/F_432_ fluorescence intensity ratios (RFU) measured in haemolymph samples collected 24 hrs after injection with either vehicle only (vehicle-vehicle), AAPH only (AAPH-vehicle), BSA only (vehicle BSA), or injection of both AAPH and BSA (AAPH-BSA). Note that timing and type of injections were identical as for the behavioral assessment depicted in Figure 6A. BSA-only injection significantly reduces F_505_/F_432_ ratio when compared with vehicle only injected animals. There is a significant increase in F_505_/F_432_ ratio after AAPH injection. This effect was eliminated in the combined AAPH-BSA treatment group. **p < 0.01.

Above we showed that injection with exogenous arachidonic acid prior to training has no effect on appetitive LTM function. However, arachidonic acid is likely not the only PLA_2_-hydrolysis product released with neuromodulatory or signaling capabilities. Defatted bovine serum albumin (BSA) is considered a high affinity FFA binding protein [28,29]. Thus, we first verified whether BSA is an effective tool for haemolymph FFA manipulation in *L. stagnalis.* Animals were injected with AAPH or vehicle-only and 24 hrs later subsequently injected with defatted BSA or vehicle-only. Two hours after BSA injections, haemolymph was collected and FFA levels were determined immediately using the ADIFAB assay. As before, AAPH injection induced elevated haemolymph FFA (Figure 5; One-way ANOVA F_3,19_ = 23.695, p < 0.0001; vehicle vs. AAPH F_1,19_ = 16.522, p = 0.0006). Moreover our data showed that BSA effectively binds FFAs in *L. stagnalis* haemolymph. That is, naturally occurring FFAs levels are significantly lower after BSA injection (vehicle vs BSA F_1,19_ = 19.385, p = 0.0003) and animals subjected to injections of both AAPH and BSA have similar haemolymph FFA levels as vehicle-only injected animals (vehicle vs AAPH-BSA F_1,19_ = 1.519, p = 0.23).

**Figure 6:**
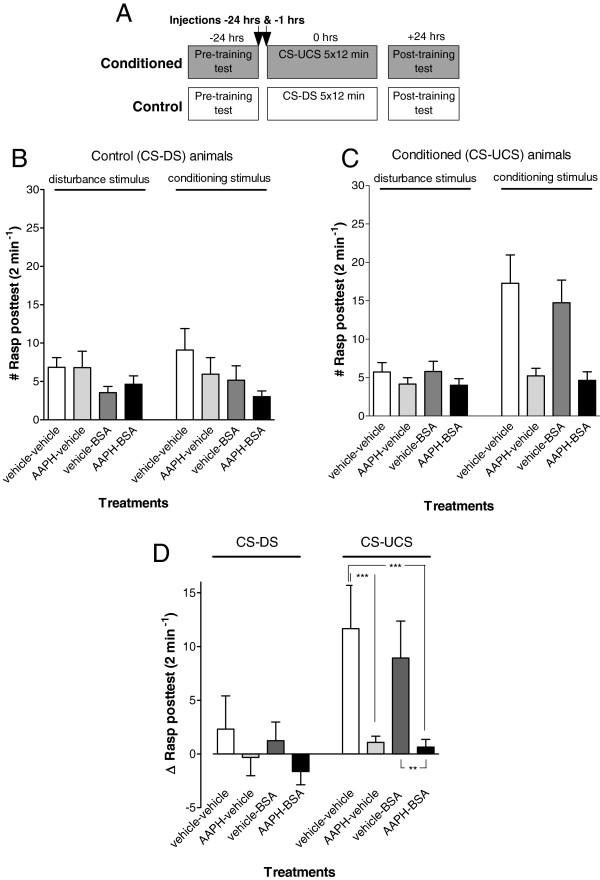
**Sequestering circulating FFAs with BSA does not restore LTM failure in AAPH injected animals. A**. Protocol indicating timing of injection and start of pre- and post-training tests with respect to the start of the first training session for both the conditioned (CS-UCS) and control (CS-DS) animals. **B-D**. LTM assessment in animals treated with vehicle-only (vehicle-vehicle), AAPH only (AAPH-vehicle), BSA only (vehicle-BSA) or injections of both AAPH and BSA (AAPH-BSA). In all control animals, rasping movements were similar upon application of the disturbance stimulus or conditioning stimulus **(B)**. In contrast, conditioned animals, either vehicle injected or BSA only injected, showed an increase in the number of rasping movements after application of the conditioning stimulus **(C)**. Thus, both the AAPH-only and AAPH-BSA injected conditioned groups, showed a significant reduction in their Δrasp values in the post-training test compared to the other conditioned groups. Animals injected with aristolochic acid only were not different in their conditioned response than the vehicle injected animals **(D)**. This suggests that co-treatment of AAPH with BSA, does not reverse the AAPH induced adverse effect on appetitive LTM performance. **p < 0.01; ***p < 0.001.

Next we tested whether BSA-injection could reverse AAPH induced appetitive LTM failure. Animals were injected with AAPH or vehicle-only 24 hrs before training and subsequently injected 1 hr before training with defatted BSA or vehicle (Figure 6A; note that the injection regime was identical as used for the previous experiment). We found that injection with BSA did not counter AAPH’s negative effect on LTM formation (Figure 6). As before, treatment with AAPH caused a significant reduction in the conditioned feeding response compared to vehicle treated conditioned animals (ANOVA interaction training x treatment, F_3,102_ = 2.748, p < 0.05; vehicle vs AAPH F_1,102_ = 8.7611, p = 0.003). In contrast, animals treated with BSA-only learned well (F_1,102_ = 13.805, p = 0.0003) in a manner that was statistically indistinguishable from that of vehicle treated conditioned animals (Figure 6B; F_1,102_ = 0.0949, p = 0.76). However, animals treated with the combination of AAPH + BSA did not display a significant conditioned feeding response (Figure 6B black bars; F_1,102_ = 0.5285, p = 0.47). This conditioned feeding response was different compared to vehicle or BSA treated conditioned animals (F_1,102_ = 12.717, p < 0.0001 and F_1,102_ = 10.26107, p = 0.002 for vehicle and BSA respectively) but not significantly different from the AAPH-only injected animals (Figure 6B; F_1,102_ = 0.0183, p = 0.89).

## Discussion

This study’s primary purpose was to test the hypothesis that FFAs released by oxidative stress-induced PLA_2_ activity are a factor in learning and memory dysfunction in *L. stagnalis.* Our primary observations are: 1- Increasing the level of extracellular PLA_2_ activity through injection of venom-derived extracellular PLA_2_ evoked LTM impairment. 2- Experimental induction of oxidative stress with the lipid peroxidation-inducing free radical generator AAPH triggers an increase in circulating FFA levels that peaks within 24–48 hrs and attenuates LTM performance. 3- Experimental elevation of circulating arachidonic acid through intracoelomic injection of exogenous arachidonic acid does not affect LTM performance. 4. Experimentally induced oxidative stress-associated LTM impairment can be rescued with the general PLA_2_ inhibitor aristolochic acid but not by normalizing the level of circulating FFAs with defatted BSA.

The present data substantiate previous evidence where we link lipid peroxidation and PLA_2_ activation to age- and oxidative stress-associated LTM impairment in another, aversive operant conditioning learning and memory paradigm [7]. In both our learning models age- and oxidative stress associated LTM failure is associated with reduced electrical excitability of key interneurons in the circuits underlying the respective behaviours [6,7,22,30]. Both behavioural and electrophysiological facets of age-associated respiratory and appetitive LTM impairment can be reproduced in young animals through treatment with AAPH, a water-soluble free radical generator commonly used to induce FA peroxidation (present study and [7]). In addition, in both our two models, all behavioural, electrophysiological and biochemical symptoms of aging and experimental oxidative stress can be reversed by treatment with aristolochic acid, a broad spectrum PLA_2_ inhibitor (present study and [6,7,11]). No evidence was found of significant age-associated impairment or experimental oxidative stress-induced repression of short/intermediate term forms in either of the two behavioural conditioning paradigms [7,27], suggesting that transcription-independent forms of memory are relatively impervious to aging or oxidative stress. Intriguingly, selective appetitive LTM impairment could also be induced in young *Lymnaea* through systemic activation of their cellular immune system and, as before in aged and oxidation-stressed young animals, LTM could be rescued by means of PLA_2_ inhibition [22]. Thus, although, the possibility exists that the cellular and molecular mechanisms underlying oxidative stress induced PLA_2_ activation dependent LTM impairments in our two learning models are different, this seems unlikely. The brain and its neurons are due to their high polyunsaturated fatty acids (PUFA) content inordinately sensitive to free radical attack, oxidative stress and subsequent peroxidation, a process that if not properly contained can severely disrupt membrane architecture and lipid signaling processes [31-33]. Neurons defend themselves against lipid-peroxidation through various mechanisms, one that involves excision of (per)oxidized FA by PLA_2_[12,15]. As will be discussed below, PLA_2_, (per)oxidized FA and their various metabolites all can alter signal transduction, various neuronal signaling pathways, ion channel functioning and gene transcription processes and ultimately behavioral plasticity such as learning and memory processes. Thus, although to our knowledge our studies are the first to show these aspects in molluscs, it is not surprisingly that similar conclusions are drawn with increasing frequency in studies linking oxidative stress related PLA_2_ activation with cognitive impairment, neuronal dysfunction and disease not only in other, non-molluscan, invertebrate species but also in mammals and humans [12,16,34-38]. Together this suggests that *Lymnaea’s* LTM functions in general are sensitive to lipid peroxidation and oxidative membrane damage, and that lipid peroxidation-dependent activation of PLA_2_ is a fundamental and evolutionary conserved factor in the decline in behavioral and neuronal plasticity widely observed across the animal kingdom.

Activation of PLA_2_ by reactive oxygen species in *L. stagnalis* results in the release of extracellular (per)oxidized FFAs (present study and [7]). Many FFAs, including AA and DHA the two FAs most commonly found at the glycerophospholipid *sn-2* position in neurons, have various demonstrated biological activity in the nervous system. For instance, under normal conditions they can regulate membrane fluidity and other aspects of phospholipid membrane microarchitecture, may engage in modulatory interactions with various ion channels, affect alterations in membrane protein clustering, modify receptor sensitivity and signal transduction pathways as well as affect gene transcription processes [17-21,39-43]. However, under conditions of oxidative stress, causes (over) activation of PLA_2_ resulting in peroxidized FA excision from the lipid bilayer matrix [12,15]. Recent evidence increasingly associates deregulation of lipid metabolism due to PLA_2_ (over) activation as a cause of nervous system dysfunction and cognitive impairment [7,12,22,23]. In addition, AA is the substrate for many potentially neuroactive lipids metabolized including those generated by the eicosanoid metabolic pathways. Each of the three main branches of eicosanoid metabolism, the epoxygenase or CYP-450, the lipoxygenase (LOX) and the cyclooxygenase (COX) pathways, has the potential to generate a variety of lipid-derived neuromodulatory substances that may engage with either intracellular or extracellular targets [16,18,20,41,44]. In addition, (per)oxidized FFAs can undergo further metabolic processing resulting in the production of highly cytotoxic aldehydes such as 4-hydroxynonenal and malondialdehyde [1,14].

Interestingly, we recently showed that challenging the immune system impairs LTM function in *L. stagnalis* that seems to involve PLA_2_ and COX activity [22]. In addition, non-enzymatic lipid peroxidation (including AAPH induced), has been shown to result in the production of AA and DHA derived iso- and neuroprostanes including the LOX series [45,46]. PLA_2_- and/or LOX-dependent arachidonic-acid modulated background K^+^ channels have been described in *Lymnaea* and various other molluscs [47-51]. Opening of these 4TM2P channels generates an outward current. It is therefore conceivable that elevated levels of PLA_2_-activity associated with inflammation, oxidative stress and aging induce a decline in neuronal excitability through activation of these 4TM2P background K^+^ channels. In this respect the 12-LOX product 12-HPETE, first described in the gastropod *Aplysia californica*, is quite interesting. 12-HPETE has long been known as an activator of *Aplysia’s* S-type K^+^-channels [52]. These K^+^ channels, later identified to belong to the TREK-1 family, are instrumental in non-synaptic forms of plasticity underlying behavioral modification of *Aplysia*’s gill withdrawal reflex [53] and are implicated as synaptic modulator in *Aplysia* and various vertebrate model systems [54-58]. Alterations in receptor sensitivity, ion channels and signal transduction pathways as well as affect gene transcription by PLA_2_, it’s products and their metabolites will most likely affect activity,- transcription- and protein synthesis-dependent processes such as LTM formation.

Based on the evidence described above it seems therefore very plausible that lipid-peroxidation evoked elevated levels of FFAs through activation of PLA_2_ are the source of the observed LTM dysfunction in *L. stagnalis* (present study and [7,22]). However, in the present study we show that increasing extracellular levels of AA does not affect LTM performance in *L. stagnalis*. Moreover, we also show in the current study that removing AAPH-induced elevated levels of circulating peroxidized FFA with BSA does not rescue LTM failure in our model system.

So, if not extracellular FFAs then what might be a source of the lipid-peroxidation dependent LTM failure observed in *L. stagnalis*? The first possibility is that PLA_2_ itself is the main culprit. The PLA_2_ family is a family of heterogeneous enzymes acting in different cellular locations and to some extent different activation profiles usually classified into intracellular located cytosolic PLA_2_ (cPLA_2_), calcium-independent PLA_2_ (iPLA_2_), extracellular acting secretory PLA_2_ (sPLA_2_) and the recently identified lysosomal and adipose-specific PLA_2_’s [15,34,59]. Upon activation, the various PLA_2_ family members such as cPLA2 and sPLA2 can induce FA release and eicosanoid production by themselves [60-62]. In addition, recent evidence also indicates the existence of cross-talk and trans-activation between the various intracellular and cytosolic PLA_2_ family members [63-65]. Thus it is quite conceivable that AAPH, a water-soluble extracellular free radical generator used to induce lipid peroxidation in the present study activates various intracellular and extracellular PLA_2_ enzymes.

Besides being present and acting on the cellular plasmamembrane both cPLA_2_ and iPLA_2_ can localize and/or target organellar membranes including mitochondrial and nuclear membranes [34,66-68]. For instance, it has been shown that activation of PLA_2_ can induce mitochondrial dysfunction due to a loss of mitochondrial membrane potential, swelling of mitochondria and/or the production of superoxide from mitochondria [69]. Furthermore, interactions between cPLA_2_ and NADPH-oxidase complexes in the plasmamembrane have been implicated in the redox-pathology of various neurodegenerative diseases [34,70]. In addition to their potential interference with numerous signaling processes, PLA_2_ enzymatic activity may impact various membrane architecture-sensitive processes. For example, PLA_2_ may alter phospholipid-packing causing membranes to become more molecularly ordered and affect processes involved in vesicle fusion, exocytosis and actin-dependent processes [71-75]. Moreover, there is evidence that architectural changes induced by PLA_2_ may alter gating states for a variety of ion channels including members of the K2P two-pore “leak” potassium family [76,77]. At this point in time we cannot say if and which of these scenarios underpins LTM impairment in our model system. Nor can we pinpoint which PLA_2_ is involved. However, the observation in the current and one of our previous studies [7], that treatment with aristolochic acid, commonly considered a general PLA_2_ inhibitor and inhibiting various classes of PLA_2_[68,78-81], reverses the AAPH-induced LTM failure is consistent with PLA_2_’s involvement.

Another intriguing potential scenario to consider as explanation for the current results is the possibility that other products released extracellular after PLA_2_ activation are causing LTM failure in *L. stagnalis*. For instance, hydrolysis of oxidized membrane phospholipids by PLA_2_ will not only liberate FFAs but also produce lysophospholipids (LPLs). Involvement of LPLs in membrane-associated processes such as membrane budding, ruffle formation, protein complex assembly and ion channel gating has been reported [34,59,82-85]. However, extracellular LPLs, like FFAs, can bind to BSA [86-88]. Therefore, we currently interpret BSA’s inability to reverse AAPH-induced LTM deficiency as evidence against the idea that circulating LPLs play a major role in the current LTM failure model. Alternatively, extracellular (per)oxidized FAs can undergo further metabolic processing resulting in the production of highly cytotoxic aldehydes such as 4-hydroxynonenal and malondialdehyde [1,14]. It is important to note that BSA is not cell permeable. Thus, indirect activation of intracellular PLA_2_ might cause elevated levels of intracellular FFAs and LPLs thereby affecting various intracellular targets [14,89,90]. For instance, it has been demonstrated that increased levels of intracellular FFAs can directly affect NADPH-oxidase [90,91] resulting in a further increase of ROS and lipid peroxidation [91].

In addition to FFAs and LPLs, PLA_2_ activation results in the extracellular release of lyso-platelet-activating factor (lyso-PAF), the platelet-activating factor (PAF) precursor [92-94]. PAF is a bioactive phospholipid that under normal conditions is thought to be involved in the regulation of synaptic plasticity, memory and neuronal protection [92-94]. In addition, PAF is a transcriptional activator of the cyclooxygenase-2 (COX-2) gene [92]. As noted before, AA is the primary substrate for the COX branch of the eicosanoid (inflammatory) pathway. Importantly, ROS can also be generated as a by-product of COX activity, thus creating a positive feedback loop that potentially can cause escalation of PLA_2_, PAF and COX-activity thereby causing substantive deregulation of lipid homeostasis [92-94]. In this respect it is interesting to note that we recently showed that systemic immune challenges in *L. stagnalis* induces a PLA_2_-dependent LTM failure that could be rescued with treatment of indomethacin, a putative COX inhibitor [22].

One of the intriguing questions still remaining is why inhibition of PLA_2_ 24 hrs after its activation is sufficient to reverse the oxidative stress induced LTM failure? We show in the present study that circulating FFA levels are their highest level 24–48 hrs after PLA_2_ activation suggesting that some of the PLA_2_ and FFA-dependent and associated pathways are initiated in the first 24 hrs. after induction of oxidative stress. At present we cannot definitively provide a mechanism to what causes the PLA_2_-dependent LTM failure within this time frame. However based on the actions of PLA_2_, FFA and their various products as outlined above there are some potential explanations. For instance, it is conceivable that by inhibiting PLA_2_ activity, even though FFAs are already released for some time, we stop the positive feedback loop discussed above, thereby preventing further escalation of PLA_2_, its products and their pathways. Notwithstanding, further investigations are clearly needed to resolve this issue.

## Conclusion

The current results support our earlier work where we link oxidative stress, lipid metabolism and age-associated LTM impairment in *L. stagnalis*. In the present study, we provide evidence that lipid peroxidation induced elevated levels of circulating FFA do not impact LTM performance. This is of particularly interest considering the increasing focus on the interplay between ROS, PLA_2_, COX, their substrates and metabolites as a potential factor in the deregulation of lipid metabolism and cell- and neuronal dysfunction and cognitive performance.

## Methods

### Animals

Animals were bred and raised under constant and strictly controlled ambient conditions as previously described [6,7,11,22,27,30,95]. Water used in the facility was sourced from a reverse osmosis system and reconditioned to a conductivity of ~450 Ω.cm through the addition of Instant Ocean salts at 1 g/US Gallon (i.e., artificial pond water; Aquarium Systems USA). Calcium concentration was kept at saturation level (~60 mg/L as CaCO_3_) through the addition of calcium carbonate (light powder; EMD analytics, Gibbstown, New Jersey) to the tanks. In addition, animals had continuous access to sterilized cuttlefish (*Sepia officinalis*) bone (2–3 per tank). Animals were fed a routine diet consisting of Romaine lettuce and Aquamax-carniverous Grower 600 trout pellets *ad libitum* (Purina Mills LLC, St. Louis, Missouri). For the present study, young sexually mature snails (age 7–9 months; shell length 2.5 cm-3 cm; see also [7]) were taken at random from healthy populations. The use and care of animals conformed to the University of Calgary Animal Care and Use Policy which adheres to the guidelines, policies and standards of the Canadian Council on Animal Care (CCAC), the Canadian Association of Laboratory Animal Medicine (CALAM), standards of Veterinary Care, and the Alberta Veterinary Association (AVMA) professional codes and standards.

### Training and testing procedures

#### Preparation

Behavioural conditioning was done using a non-aversive appetitive classical conditioning protocol [22,27]. Snails were sampled at random and marked, for identification purposes with indelible marker. Food was withheld starting 48 hrs prior to the first pre-training test and for the remainder of the training and post-training testing.

#### Testing procedures

On day 1, prior to behavioural conditioning, each snail was individually tested for their natural response to the administration of pond water, the disturbance stimulus (DS), as well as the conditional stimulus (CS) n-amyl acetate (“Pre-training test”) [22]. Tests were performed using 100-ml translucent polystyrene beakers (4.5 cm diameter), filled with 80 mL of water taken from the snail’s home tank. After transfer into the beakers, the snails were allowed to acclimatize for 15 min before testing commenced. Testing involved counting the number of rasps over two consecutive periods of 2 min, the first period starting with gentle administration of the DS (10 ml artificial pond water), the second period starting with the administration of the CS (10 ml n-amyl acetate solution; 4 ppm final concentration). To facilitate observation, the test beakers were elevated by translucent plastic stands and were surrounded by mirrors to ensure continuous view of the buccal mass and radula of the snails during testing. A test response was calculated by taking the difference between the number of rasps counted during the second period minus the number counted during the first period (i.e., ΔRasp = rasps after CS – rasps after DS). To correct for both differences in background rasping activity and potential application artefacts, the pre-training tests were performed in duplicate with >1 hr interval, and behavioural responses were calculated as the average of both ΔRasp (i.e., ΔRasp_pre-test_ = average ΔRasp_pre-test_1 and ΔRasp_pre-test_2). After completion of a test, snails were gently rinsed and returned to their home tanks. A single post-training test was performed on day 3 (22–24 hrs after training) following identical procedures as described for the pre-testing above.

#### Training procedure

Snails were trained in a single day, multi-trial forward-delay conditioning format [22]. Sucrose (final concentration of 0.4% wt/vol) served as the unconditioned stimulus (UCS) and n-amyl acetate (4 ppm final concentration) as the conditioned stimulus (CS). To control for potential behavioural effects of fluid addition, a disturbance control in which the UCS was paired with the DS (i.e., pond water) was implemented. Snails were randomly assigned to either the CS–UCS (“conditioned”) or the CS–DS (unconditioned “control”) group and trained “en masse”. Training was performed in 1-L polypropylene beakers containing 480 ml clean artificial pond water. After transfer into the training beakers, the snails were allowed to acclimatize for 60 min. Both “control” and “conditioned” groups received 120 mL of the CS solution, followed 15 s later by 120 ml of the UCS (“conditioned” group) or 120 ml of the DS (“control” group). After 2 min, the beakers containing the snails were drained and gently rinsed with clean pond water and the snails were readied for their next training trial by re-placing them in the 1-L polypropylene beakers holding 480 ml clean artificial pond water. After 11 min and 45 sec the training procedure was repeated. Snails received a total of 5 training procedures on a single day before being returned to their “home” tank. Snails were at all times fully submerged during training and testing. Care was taken to ensure that pre-testing, training and post-testing commenced at the same time of day for each group and training and testing always occurred in the same location. “Conditioned” and “control” snails were always tested and trained concurrently.

### Chemicals and drug injections

All chemicals used in this study i.e., defatted bovine albumin serum (BSA; fatty acid free), aristolochic acid, arachidonic acid, Phospholipase A_2_ from honey bee venom and 2,2-azobis (2-methylpropion-amidine) dihydrochloride (AAPH) were obtained from Sigma Aldrich (St. Louis, MO). All drugs, except arachidonic acid, were dissolved in sterile ultrapure water. Arachidonic acid was dissolved in dimethyl sulfoxide (DMSO). To prevent premature oxidation of dissolved AA, the drug was either used immediately after dissolving or stored under nitrogen at −20°C. Drug or vehicle-only treatments were delivered by means of intracoelomic injection into the snail (note: this will introduce drugs directly into the animal’s blood and circulatory system) in a manner avoiding whole-body withdrawal responses and voiding of haemolymph [7,22,30]. Using body weight measurements injected amounts of drugs were calculated to achieve the following approximate concentrations of drugs in the snails’ haemolymph: 1 mM of AAPH, 2 mg/ml of BSA, 20U of PLA_2_, 10 μM of both arachidonic acid and aristolochic acid. Haemolymph DMSO concentrations never exceeded 0.05%. As control snails were injected with equivalent volumes vehicle only (vehicle control). Except for AAPH injected 24 hrs before training, all other injections were given 1 hour prior to the start of the first training trial. AAPH is a water-soluble slowly dissociating azo-compound used extensively as a free radical generator in studies of lipid peroxidation and characterization of antioxidants in various model systems including *L. stagnalis*[6,7,96,97]. All solutions were injected through the foot directly into the haemocoel with a microliter syringe and a 25G needle. Animals were behaving normally within 1–2 minutes after injection.

### Haemolymph extraction and free fatty acid detection

Snails were injected with AAPH, BSA, AAPH + BSA or vehicle-only using the same injection schedule as for the behavioural assays. Haemolymph was extracted by head–foot retraction [98]. Haemolymph collected of 3 snails/condition was pooled. Free fatty acid levels were quantified using standard protocols involving acrylodan labeled intestinal fatty acid binding protein (ADIFAB; FFA Sciences, San Diego, CA) [7,99,100]. Haemolymph plus ADIFAB (0.2 μM) was loaded on a 96-well plate. Fluorescent signal was measured immediately using a Spectromax 2Me multidetection microplate reader (Molecular Devices, Sunnyvale, CA) with and excitation wavelength of 386 nm and emission wavelength of 432 and 505 nm representing respectively unbound and bound states of ADIFAB. Data are expressed in dimensionless fluorescence intensity units (RFU) determined from intensity ratios measured at 505 and 432 nm (F_505_/F_432_; see [99]). An increased F_505_/F_432_ ratio is indicative of increased haemolymph free fatty acid levels [99,100]. Six or more independent experiments with each 3 replicates/condition were performed.

### Statistical analysis

Behavioural data was analyzed by means of a factorial or repeated measure ANOVA. Explicit hypotheses were tested using planned comparisons unless indicated differently in the text. Effect of drugs on FFA production was analyzed by one-way ANOVA. Compliance with parametric assumptions was confirmed for each data set submitted to ANOVA using both graphical (probability plots applied to raw data and residuals) and analytical techniques (Kolmogorov-Smirnov one-sample test for normality, F-max test). Throughout the text, average and data dispersion are expressed as arithmetic means and standard error of the mean (SEM). Figures were constructed using Graphpad Prism version 4.03 (Graphpad Sofware Inc., La Jolla, CA).

## Abbreviations

BSA: Bovine serum albumin; CNS: Central nervous system; COX: Cyclooxygenase; CS: Conditioned stimulus; DMSO: Dimethyl sulfoxide; DS: Disturbance stimulus; FFA: Free fatty acid; HBS: Hepes buffered saline; LTM: Long-term memory; PAF: Platelet-activating factor; PLA2: Phospholipase A2; ROS: Reactive oxygen species; UCS: Unconditioned stimulus.

## Competing interests

The authors declare that they have no competing interests.

## Authors’ contributions

Conceived research program WCW. Conceived and designed the experiments: PMH, WCW. Performed the experiments: EB, JI, SNW, PMH. Analyzed the data EB, JI, SNW, PMH, WCW. Wrote the paper: EB, PMH, WCW. All authors read and approved the final manuscript.

## References

[B1] Negre-Salvayre A Auge N Ayala V Basaga H Boada J Brenke R Chapple S Cohen G Feher J Grune T Lengyel G Mann GE Pamplona R Poli G Portero-Otin M Riahi Y Salvayre R Sasson S Serrano J Shamni O Siems W Siow RC Wiswedel I Zarkovic K Zarkovic N Pathological aspects of lipid peroxidation Free Radical Res 2010 15 1125 1171 10.3109/10715762.2010.498478 20836660

[B2] Niki E Do antioxidants impair signalling by reactive oxygen species and lipid peroxidation products FEBS Lett 2012 15 3767 3770 10.1016/j.febslet.2012.09.025 23022561

[B3] Kelly A Vereker E Nolan Y Brady M Barry C Loscher CE Mills KHG Lynch MA Activation of p38 plays a pivotal role in the inhibitory effect of lipopolysaccharide and interleukin-1 beta on long term potentiation in rat dentate gyrus J Biol Chem 2003 15 19453 19462 10.1074/jbc.M301938200 12609991

[B4] Massaad CA Klann E Reactive oxygen species in the regulation of synaptic plasticity and memory Antioxid Redox Signal 2011 15 2013 2053 10.1089/ars.2010.3208 20649473 PMC3078504

[B5] Stranahan AM Mattson MP Recruiting adaptive cellular stress responses for succssful brain aging Nat Rev 2012 15 209 216 10.1038/nrn3151 PMC4084510 22251954

[B6] Watson SN Nelson MA Wildering WC Redox agents modulate neuronal activity and reproduce physiological aspects of neuronal aging Neurobiol Aging 2012 15 1 149 161 10.1016/j.neurobiolaging.2010.01.017 20153084

[B7] Watson SN Wright N Hermann PM Wildering WC Phospholipase A2: The key to reversing long-term memory impairment in a gastropod model of aging Neurobiol Aging 2013 15 2 610 620 10.1016/j.neurobiolaging.2012.02.028 22459601

[B8] Cini M Moretti A Studies on lipid peroxidation and protein oxidation in the aging brain Neurobiol Aging 1995 15 1 53 57 10.1016/0197-4580(95)80007-E 7723936

[B9] Catala A A synopsis of the process of lipid peroxidation since the discovery of the essential fatty acids Biochem Biophys Res Commun 2010 15 3 318 323 10.1016/j.bbrc.2010.07.087 20674543

[B10] Spiteller G Is lipid peroxidation of polyunsaturated acids the only source of free radicals that induce aging and age-related diseases? Rejuv Res 2010 15 1 91 103 10.1089/rej.2009.0934 20230283

[B11] Watson SN Lee JR Risling TE Hermann PM Wildering WC Diminishing GSH availability and age-associated decline in neuronal excitability Neurobiol Aging 2014 15 5 1074 1085 10.1016/j.neurobiolaging.2013.11.007 24331753

[B12] Adibhatla RM Hatcher JF Phospholipase A2, reactive oxygen species and lipid peroxidation in CNS pathologies BMB Rep 2008 15 8 560 567 10.5483/BMBRep.2008.41.8.560 18755070 PMC2920609

[B13] Goldman R Ferber E Zort U Reactive oxygen species are involved in the activation of cellular phospholipase A2 FEBS Lett 1992 15 190 192 10.1016/0014-5793(92)81092-Z 1505682

[B14] Farooqui AA Horrocks LA Phospholipase A2-generated lipid mediators in the brain: the good, the bad and the ugly Neuroscientist 2006 15 245 260 10.1177/1073858405285923 16684969

[B15] Murakami M Taketomi Y Miki Y Sato H Hirabayashi T Yamamoto K Recent progress in phospholipase A2 research: from cells to animals to humans Prog Lipid Res 2011 15 152 192 10.1016/j.plipres.2010.12.001 21185866

[B16] Phillis JW Horrocks LA Farooqui AA Cyclooxygenases, lipoxygenases, and epoxygenases in CNS: Their role and involvement in neurological disorders Brain Res Rev 2006 15 201 243 10.1016/j.brainresrev.2006.02.002 16647138

[B17] Meves H The action of prostaglandins on ion channels Curr Neuropharm 2006 15 41 57 10.2174/157015906775203048 PMC2430679 18615137

[B18] Meves H Arachidonic acid and ion channels: an update Brit J Pharmacol 2008 15 4 16 10.1038/bjp.2008.216 18552881 PMC2527843

[B19] Ordway RW Singer JJ Walsh JV Jr Direct regulation of ion channels by fatty acids TINS 1991 15 3 96 100 1709540 10.1016/0166-2236(91)90069-7

[B20] Piomelli D Eicosanoids in synaptic transmission Crit Rev Neurobiol 1994 15 1/2 65 83 8124731

[B21] Tassoni D Kaur G Weisinger RS Sinclair AJ The role of eicosanoids in the brain Asia Pac J Clin Nutr 2008 15 1 220 228 18296342

[B22] Hermann PM Park D Beaulieu E Wildering WC Evidence for inflammation-mediated memory dysfunction in gastropods: putative PLA2 and COX inhibitors abolish long-term memory failure induced by systemic immune challenges BMC Neurosci 2013 15 83 10.1186/1471-2202-14-83 23915010 PMC3750374

[B23] Sanchez-Mejia RO Mucke L Phospholipase A2 and arachidonic acid in Alzheimer’s disease Biochim Biophys Acta 2010 15 8 784 790 10.1016/j.bbalip.2010.05.013 20553961 PMC3024142

[B24] Alexander JE Audesirk TE Audesirk GJ Rapid, nonaversive conditioning in a freshwater gastropod. II. Effects of termporal relationships on learning Behav Neural Biol 1982 15 4 391 402 10.1016/S0163-1047(82)90792-0 7184501

[B25] Audesirk TE Alexander JE Audesirk GJ Moyer CM Rapid, nonaversive conditioning in a freshwater gastropod. I. Effects of age and motivation Behav Neural Biol 1982 15 4 379 390 10.1016/S0163-1047(82)90782-8 7184500

[B26] Benjamin PR Staras K Kemenes G A systems approach to the cellular analysis of associative learning in the pond snail Lymnaea Learn Mem 2000 15 3 124 131 10.1101/lm.7.3.124 10837501

[B27] Hermann PM Lee A Hulliger S Minvielle M Ma B Wildering WC Impairmtent of long-term associative memory in aging snails (Lymnaea stagnalis) Behav Neurosci 2007 15 6 1400 1414 18085894 10.1037/0735-7044.121.6.1400

[B28] McArthur MJ Atshaves BP Frolov A Foxworth WD Kier AB Schroeder F Cellular uptake and intracellular trafficking of long chain fatty acids J Lipid Res 1999 15 1371 1383 10428973

[B29] Richieri GV Ogata RT Kleinfeld AM Kinetics of fatty acid interactions with fatty acid binding proteins from adipocyte, heart, and intestine J Biol Chem 1996 15 11291 11300 10.1074/jbc.271.19.11291 8626681

[B30] Watson SN Risling TE Hermann PM Wildering WC Failure of delayed nonsynaptic neuronal plasticity underlies age-associated long-term associative memory impairment BMC Neurosci 2012 15 113 10.1186/1471-2202-13-113 22898271 PMC3470963

[B31] Forlenza OV Schaeffer EL Gattaz WF The role of phospholipase A2 in neuronal homeostasis and memory formation: implications for the pathogenesis of Alzheimer’s disease J Neural Transm 2007 15 2 231 238 10.1007/s00702-006-0597-0 17131232

[B32] Currais A Maher P Functional consequences of age-dependent changes in glutathione status in the brain Antioxid Redox Signal 2013 15 8 813 822 10.1089/ars.2012.4996 23249101

[B33] Ballatori N Krance SM Notenboom S Shi S Tieu K Hammond CL Glutathione dysregulation and the etiology and progression of human diseases Biol Chem 2009 15 191 214 19166318 10.1515/BC.2009.033 PMC2756154

[B34] Lee JV-M Simonyi A Sun AY Sun GY Phospholipase A2 and neural membrane dynamics: Implications for Alzheimer’s disease J Neurochem 2011 15 813 819 10.1111/j.1471-4159.2010.07033.x 21214562 PMC3058290

[B35] Farooqui T Iron-induced oxidative stress modulates olfactory learning and memory in honeybees Behav Neurosci 2008 15 433 447 18410182 10.1037/0735-7044.122.2.433

[B36] Murakami S Caenorhabditis elegans as a model system to study aging of learning and memory Molec Neurobiol 2007 15 85 93 10.1007/BF02700625 17519507

[B37] Jaiswal M Sandoval H Zhang K Bayat V Bellen HJ Probing mechanisms that underlie human neurodegenerative diseases in Drosophila Annu Rev Genet 2012 15 371 396 10.1146/annurev-genet-110711-155456 22974305 PMC3663445

[B38] Sestini EA Carlson JC Allsopp R The effects of ambient temperature on life span, lipid peroxidation, suproxide dismutase, and phospholipase A2 activity in Drosophila melanogaster Exp Gerontol 1991 15 385 395 10.1016/0531-5565(91)90050-V 1936197

[B39] Bruno MJ Koeppe RE Andersen OS Docosahexaenoic acid alters bilayer elastic properties Proc Natl Acad Sci 2007 15 9638 9643 10.1073/pnas.0701015104 17535898 PMC1887599

[B40] Chapkin RS Wang N Fan YY Lupton JR Prior IA Docosahexaenoic acid alters the size and distribution of cell surface microdomains Biochim Biophys Acta 2008 15 466 471 10.1016/j.bbamem.2007.11.003 18068112 PMC2244794

[B41] Di Marzo V Arachidonic acid and eicosanoids as targets and effectors in second messenger interactions Prostaglandins Leukot Essent Fatty Acids 1995 15 4 239 254 10.1016/0952-3278(95)90123-X 8577777

[B42] Stillwell W Shaikh SR Zerouga M Siddiqui R Wassal SR Docosahexaenoic acid affects cell signaling by altering lipd rafts Reprod Nutr Dev 2005 15 559 579 10.1051/rnd:2005046 16188208

[B43] Yang X Sheng W Sun GY Lee JC Effects of fatty acid unsaturation numbers on membrane fluidity and a-secretase-dependent amyloid precursor protein processing Neurochem Int 2011 15 321 329 10.1016/j.neuint.2010.12.004 21184792 PMC3040984

[B44] Reilly MP Lawson JA FitzGerald GA Eicosanoids and isoeicosanoids: indices of cellular function and oxidant stress J Nutr 1998 15 2 Suppl 434S 438S 9478043 10.1093/jn/128.2.434S

[B45] Montine KS Quinn JF Zhang J Fessel JP Roberts LJ Morrow JD Montine TJ Isoprostanes and related products of lipid peroxidation in neurodegenerative diseases Chem Phys Lipids 2004 15 1–2 117 124 15037157 10.1016/j.chemphyslip.2003.10.010

[B46] Roberts LJ Fessel JP The biochemistry of the isoprostane, neuroprostane, and isofuran pathways of lipid peroxidation Chem Phys Lipids 2004 15 1–2 173 186 15037162 10.1016/j.chemphyslip.2003.09.016

[B47] Kits KS Lodder JC Veerman MJ Phe-Met-Arg-Phe-amide activates a novel voltage-dependent K + current through a lipoxygenase pathway in molluscan neurones J Gen Physiol 1997 15 5 611 628 10.1085/jgp.110.5.611 9348332 PMC2229393

[B48] Bahls FH Richmond JE Smith WL Haydon PG A lipoxygenase pathway of arachidonic acid metabolism mediates FMRFamide activation of a potassium current in an identified neuron of Helisoma Neurosci Lett 1992 15 1 165 168 10.1016/0304-3940(92)90497-U 1407658

[B49] Lopes CMB Franks NP Lieb WR Actions of general anaesthetics and arachidonic acid pathway inhibitors on K + currents activated by volatile anaesthetics and FMRFamide in molluscan neurones Brit J Pharm 1998 15 2 309 318 10.1038/sj.bjp.0702069 PMC1565621 9786503

[B50] Pivovarov AS Drozdova EI Zabolotskii DA Myagkova GI Eicosapolyynoic acids, inhibitors of lipoxygenases, weaken the short-term plasticity of cholinoreceptors of neurons of the edible snail Neurosci Behav Physiol 1993 15 2 176 181 10.1007/BF01189115 7683775

[B51] Pivovarov AS Egido-Villareal W The influence of an inhibitor of lipoxygenases on the modulation of the plasticity of cholinoreceptors by 15-HETE Neurosci Behav Physiol 1995 15 6 483 487 10.1007/BF02359276 8848081

[B52] Piomelli D Volterra A Dale N Siegelbaum SA Kandel ER Schwartz JH Belardetti F Lipoxygenase metabolites of arachidonic acid as second messengers for presynaptic inhibition of Aplysia sensory cells Nature 1987 15 6125 38 43 10.1038/328038a0 2439918

[B53] Hawkins RD A cellular mechanism of classical conditioning in Aplysia J Exp Biol 1984 15 113 128 6392464 10.1242/jeb.112.1.113

[B54] Schaad NC Maistretti PJ Schorderet M Prostanoids and their role in cell-cell interactions in the central nervous system Neurochem Int 1991 15 303 322 10.1016/0197-0186(91)90161-6 20504706

[B55] DeCostanzo AJ Voloshyna I Rosen ZB Feinmark SJ Siegelbaum SA 12-Lipoxygenase regulates hippocampal long-term potentiation by modulating L-type Ca2+ channels J Neurosci 2010 15 5 1822 1831 10.1523/JNEUROSCI.2168-09.2010 20130191 PMC2835505

[B56] Feinmark SJ Begum R Tsvetkov E Goussakov I Funk CD Siegelbaum SA Bolshakov VY 12-lipoxygenase metabolites of arachidonic acid mediate metabotropic glutamate receptor-dependent long-term depression at hippocampal CA3-CA1 synapses J Neurosci 2003 15 36 11427 11435 14673007 10.1523/JNEUROSCI.23-36-11427.2003 PMC6740529

[B57] Honore E The neuronal background K2P channels:focus on TREK1 Nat Rev Neurosci 2007 15 4 251 261 17375039 10.1038/nrn2117

[B58] Volterra A Buttner N Siegelbaum SA Direct opening of S-type K + channels of Aplysia sensory neurons by 12-lipoxygenase metabolites Adv Prostaglandin Thrombox Leukot Res 1991 15 727 730 1847573

[B59] Farooqui AA Horrocks LA Brain phospholipase A2: a perspective on the history Prostaglandins Leukot Essent Fatty Acids 2004 15 161 169 10.1016/j.plefa.2004.03.004 15253885

[B60] Saiga A Uozumi N Ono T Seno K Ishimoto Y Arita H Shimizu T Hanasaki K Group X secretory phospholipase A2 can induce arachidonic acid release and eicosanoid production without activation of cytosolic phospholipase A2 alpha Prostaglandins Other Lipid Mediat 2005 15 1–4 79 89 15789617 10.1016/j.prostaglandins.2004.10.001

[B61] Morioka Y Saiga A Yokota Y Suzuki N Ikeda M Ono T Nakano K Fujii N Ishizaki J Arita H Hanasaki K Mouse group X secretory phospholipase A2 induces a potent release of arachidonic acid from spleen cells and acts as a ligand for the phospholipase A2 receptor Arch Biochem Biophys 2000 15 1 31 42 10.1006/abbi.2000.1977 11019817

[B62] Gijón MA Leslie CC Regulation of arachidonic acid release and cytosolic phospholipase A2 activation J Leukoc Biol 1999 15 3 330 336 10080535 10.1002/jlb.65.3.330

[B63] Han WK Sapirstein A Hung CC Alessandrini A Bonventre JV Cross-talk between cytosolic phospholipase A2 alpha (cPLA2 alpha) and secretory phospholipase A2 (sPLA2) in hydrogen peroxide-induced arachidonic acid release in murine mesangial cells: sPLA2 regulates cPLA2 alpha activity that is responsible for arachidonic acid release J Biol Chem 2003 15 26 24153 24163 10.1074/jbc.M300424200 12676927

[B64] Fonteh AN Atsumi G LaPorte T Chilton F Secretory phospholipase A2 receptor-mediated activation of cytosolic phospholipase A2 in murine bone marrow-derived mast cells J Immunol 2000 15 5 2773 2782 10.4049/jimmunol.165.5.2773 10946309

[B65] Hernández M Burillo SL Crespo MS Nieto ML Secretory phospholipase A2 activates the cascade of mitogen-activated protein kinases and cytosolic phospholipase A2 in the human astrocytoma cell line 1321 N1 J Biol Chem 1998 15 1 606 612 10.1074/jbc.273.1.606 9417122

[B66] Gaudreault SB Chabot C Gratton JP Poirier J The caveolin scaffolding domain modifies 2-amino-3-hydroxy-5-methyl-4-isoxazole propionate receptor binding properties by inhibiting phospholipase A2 activity J Biol Chem 2004 15 356 362 10.1074/jbc.M304777200 14561756

[B67] Brustovetsky T Antonsson B Jemmerson R Dubinsky JM Brustovetsky N Activation of calcium-independent phospolipase A2 (iPLA2) in brain mitochondria and release of apoptogenic factors by BAX and truncated BID J Neurochem 2005 15 980 994 10.1111/j.1471-4159.2005.03248.x 16092941

[B68] Kinsey GR McHowat J Patrick KS Schnellmann RG Role of Ca2 + −independent phospholipase A2γ in Ca2 + −induced mitochondrial permeability transition J Pharm Exp Ther 2007 15 707 715 10.1124/jpet.107.119545 17312185

[B69] Zhu D Lai Y Shelat PB Hu C Sun GY Lee JC Phospholipase A2 mediate amyloid-beta peptide-induced mitochondrial dysfunction J Neurosci 2006 15 11111 11119 10.1523/JNEUROSCI.3505-06.2006 17065451 PMC6674660

[B70] Shmelzer Z Haddad N Admon E Pessach I Leto TL Eitan-Hazan Z Hershfinkel M Levy R Unique targeting of cytosolic phospholipase A2 to plasma membranes mediated by the NADPH oxidase in phagocytes J Cell Biol 2003 15 683 692 10.1083/jcb.200211056 12913107 PMC2173789

[B71] Churchward MA Rogasevskaia T Brandman DM Khosravani H Nava P Atkinson JK Coorssen JR Specific lipids supply critical negative spontaneous curvature - An essential component of native Ca2 + −triggered membrane fusion Biophys J 2008 15 10 3976 3986 10.1529/biophysj.107.123984 18227127 PMC2367177

[B72] Dan N Safran SA Effect of lipid characteristics on the structure of transmembrane proteins Biophys J 1998 15 3 1410 1414 10.1016/S0006-3495(98)74059-7 9726942 PMC1299815

[B73] Moes M Boonstra J Regan-Klapisz E Novel role of cPLA(2)alpha in membrane and actin dynamics Cell Mol Life Sci 2010 15 1547 1557 10.1007/s00018-010-0267-0 20112044 PMC2856858

[B74] Simonsen AC Activation of phospholipase A2 by ternary model membranes Biophys J 2008 15 3966 3975 10.1529/biophysj.107.114363 18234820 PMC2367196

[B75] Zhu D Tan KS Zhang X Sun AY Sun GY Lee JC Hydrogen peroxide alters membrane and cytoskeleton properties and increases intercellular connections in astrocytes J Cell Sci 2005 15 3695 3703 10.1242/jcs.02507 16046474

[B76] Danthi S Enyeart JA Enyeart JJ Modulation of native TREK-1 and Kv1.4 K + channels by poylunsaturated fatty acids and lysophospholipids J Membr Biol 2003 15 147 164 10.1007/s00232-003-0616-0 14724761

[B77] Patel AJ Lazdunski M Honore E Lipid and mechano-gated 2P domain K(+) channels Curr Opin Cell Biol 2001 15 422 428 10.1016/S0955-0674(00)00231-3 11454447

[B78] Lindahl M Tagesson C Selective inhibition of group II phospholipase A2 by quercetin Inflammation 1993 15 573 582 10.1007/BF00914195 8225564

[B79] Vishwanath BS Fawzy AA Franson RC Edema-induced activity of phospholipase A2 purified from human synovial fluid and inhibition by aristolochic acid Inflammation 1988 15 549 561 10.1007/BF00914317 3220517

[B80] Carnevale KA Cathcart MK Calcium-independent phospholipase A2 is required for human monocyte chemotaxis to monocyte chemoattractant protein 1 J Immunol 2001 15 3414 3421 10.4049/jimmunol.167.6.3414 11544333

[B81] Denson DD Worrell RT Middleton P Eaton DD Ca^2+^ sensitivity of BK channels in GH3 cells involves cytosolic phospholipase A2 Am J Physiol Cell Physiol 1999 15 C201 C209 10.1152/ajpcell.1999.276.1.C201 9886936

[B82] Ben-Zeev G Telias M Nussinovitch I Lysophospholipids modulate voltage-gated calcium channels currents in pituitary cells; effects of lipid stress Cell Calcium 2010 15 514 524 10.1016/j.ceca.2010.04.006 20510448

[B83] Kinnunen PKJ Kaarniranta K Mahalka AK Protein-oxidized phospholipid interactions in cellular signaling for cell death: From biophysics to clinical correlations Biochim Biophys Acta 1818 15 2446 2455 10.1016/j.bbamem.2012.04.008 22542574

[B84] Nakano T Inoue I Shinozaki R Matsui M Akatsuka T Takahashi S Tanaka K Akita M Seo M Hokari S Katayama S Komoda T A possible role of lysophospholipids produced by calcium-independent phospholipase A(2) in membrane-raft budding and fission Biochim Biophys Acta 2009 15 2222 2228 10.1016/j.bbamem.2009.07.015 19643079

[B85] Shin L Cho WJ Cook JD Stemmler TL Jena BP Membrane lipids influence protein complex assembly-disassembly J Am Chem Soc 2010 15 5596 5597 10.1021/ja101574d 20373736 PMC2862647

[B86] Han M Kim Y-L Sacket SJ Kim K Kim H-L Jo J-Y Ha B-C Im D-S Effect of direct albumin binding to sphingosylphosphorylcholine in Jurkat T cells Prost Lipid Med 2007 15 174 183 10.1016/j.prostaglandins.2007.08.003 17991619

[B87] Kim Y-L Im Y-J Ha N-C Im D-S Albumin inhibits cytotoxic activity of lysophosphatidylcholine by direct binding Prost Lipid Med 2007 15 130 138 10.1016/j.prostaglandins.2006.10.006 17259079

[B88] Thumser AEA Voysey JE Wilton DC The binding of lysophospholipids to rat liver fatty acid-binding protein and albumin Biochem J 1994 15 801 806 8053904 10.1042/bj3010801 PMC1137058

[B89] Bazan NG Tu E De Turco EB R What synaptic lipid signaling tells us about seizure-induced damage and epileptogenesis Prog Brain Res 2002 15 175 185 12143339 10.1016/S0079-6123(02)35017-9

[B90] Brash AR Arachidonic acid as a bioactive molecule J Clin Invest 2001 15 1339 1345 10.1172/JCI13210 11390413 PMC209328

[B91] Kim C Kim J-Y Kim J-H Cytosolic phospholipase A2, lipoxygenase metabolites, and reactive oxygen species BMB Rep 2008 15 555 559 18755069 10.5483/bmbrep.2008.41.8.555

[B92] Bazan NG The neuromessenger platelet-activating factor in plasticity and neurodegeneration Prog Brain Res 1998 15 281 291 9932449 10.1016/s0079-6123(08)63215-x

[B93] Chen C Bazan NG Lipid signaling: sleep, synaptic plasticity, and neuroprotection Prostaglandins Other Lipid Mediat 2005 15 65 76 10.1016/j.prostaglandins.2005.07.001 16099392

[B94] Zhu T Gobeil F Jr Vazquez-Tello A Leduc M Rihakova L Bossolasco M Bkaily G Peri K Varma DR Orvoine R Chemtob S Intracrine signalling through lipid mediators and their cognate nuclear G-protein-coupled receptors: a paradigm based on PGE2, PAF, and LPA1 receptors Can J Physiol Pharmacol 2006 15 377 391 10.1139/y05-147 16902584

[B95] Hermann PM Genereux B Wildering WC Evidence for age-dependent mating strategies in the simultaneous hermaphrodite snail, *Lymnaea stagnalis* (L.) J Exp Biol 2009 15 19 3164 3173 10.1242/jeb.030031 19749110

[B96] Liu Z Yu W Liu Z Antioxidative and prooxidative effects of coumarin derivatives on free radical initiated and photosensitized peroxidation of human low-density lipoprotein Chem Phys Lipids 1999 15 125 135 10.1016/S0009-3084(99)00101-2 10701080

[B97] Niki E Free radical initiators as source of water- or lipid-soluble peroxyl radicals Methods Enzymol 1990 15 100 108 2233287 10.1016/0076-6879(90)86095-d

[B98] Sminia T Structure and function of blood and connective tissue cells of the fresh water pulmonate Lymnaea stagnalis studied by electron microscopy and enzyme histochemistry Z Zellforsch Mikrosk Anat 1972 15 4 497 526 10.1007/BF00307004 4117131

[B99] Richieri GV Kleinfeld AM Continuous measurement of phospholipase A2 activity using the fluorescent probe ADIFAB Anal Biochem 1995 15 256 263 10.1006/abio.1995.1410 7485980

[B100] Richieri GV Ogata RT Kleinfeld AM The measurement of free fatty acid concentration with the fluorescent probe ADIFAB: a practical guide for the use of the ADIFAB probe Mol Cell Biochem 1999 15 87 94 10.1023/A:1006878421990 10331662

